# Comparative evaluation of early versus delayed appendectomy in complicated appendicitis

**DOI:** 10.6026/973206300223120

**Published:** 2026-05-31

**Authors:** Pradeep Rathore, Utkarsh Rajput, Mohammed Fazal

**Affiliations:** 1 Department of General Surgery, Government Medical College, Datia, Madhya Pradesh, India; 2 Department of General Surgery, Nagda Medical College, Sheopur, Madhya Pradesh, India

**Keywords:** Complicated appendicitis, early appendectomy, delayed appendectomy, conservative management, appendicular abscess, surgical outcomes

## Abstract

Complicated appendicitis, often presenting with perforation, abscess, or appendicular mass, continues to pose a major therapeutic challenge in surgical practice. The timing of appendectomy remains controversial, with differing opinions regarding early intervention versus delayed surgery following conservative management. Therefore, it is of interest to evaluate outcomes of early and delayed appendectomy in 100 patients diagnosed with complicated appendicitis, divided equally into Group A (Early Appendectomy, n = 50) and Group B (Delayed Appendectomy, n = 50). Operative duration, post-operative complications, hospital stay and recovery profile were systematically analyzed. Thus, we show the relative benefits of both approaches, emphasizing that early appendectomy offers shorter total hospitalization, whereas delayed intervention may lower immediate operative risks in selected cases.

## Background:

Globally, appendicitis ranks high among the most prevalent causes of acute abdomen necessitating immediate surgical surgery. In the general population, it poses a lifetime risk of around 7-8% and is responsible for a considerable fraction of emergency abdominal procedures. Although uncomplicated appendicitis is typically managed with prompt appendectomy, complicated appendicitis, which includes perforation, appendicular abscess, or appendicular mass, presents a more challenging clinical scenario for surgeons. The management strategy for complicated appendicitis has evolved considerably over the past decades, particularly regarding the timing of surgical intervention [1]. Complicated appendicitis represents approximately 20-30% of all cases of appendicitis and is associated with increased morbidity due to inflammation, adhesions and localized infection. Patients may present with perforation, peritonitis, or a localized appendicular abscess, which significantly increases the complexity of surgical management. Historically, immediate surgical removal of the appendix was considered the standard treatment; however, In addition to increasing the likelihood of complications such intestinal damage, wound infection and intra-abdominal abscess development, the presence of substantial inflammation and adhesions often rendered operation technically challenging [2]. Initial conservative therapy, often called the Ochsner-Sherren regimen, has traditionally been used to treat severe appendicitis with an appendicular mass or abscess. This technique involves intravenous antibiotics, bowel rest and supportive care. Following resolution of the acute inflammatory process, interval or delayed appendectomy is usually performed several weeks later to prevent recurrence. Studies have reported that conservative management is successful in approximately 80-90% of patients, thereby avoiding the risks associated with emergency surgery in an inflamed operative field [3]. Despite the advantages of conservative management, the concept of early appendectomy for complicated appendicitis has gained increasing attention in recent years due to improvements in surgical techniques, anesthesia and perioperative care.

Early appendectomy involves performing surgery during the initial admission soon after diagnosis. Proponents of this approach argue that early intervention allows definitive treatment during a single hospital admission reduces the risk of recurrent appendicitis and eliminates the need for a second hospitalization for interval surgery [4]. Several studies have compared early appendectomy with delayed appendectomy in complicated appendicitis. Early surgery offers the benefit of immediate source control of infection, shorter overall treatment duration and reduced risk of recurrent attacks during the waiting period. However, it may be associated with higher operative difficulty due to inflamed tissues, adhesions and distorted anatomy, which can increase operative time and intraoperative complications [5]. Conversely, delayed appendectomy after initial conservative treatment allows the inflammatory process to subside, resulting in easier surgical dissection and lower intraoperative complications. Nevertheless, this approach may require longer total hospital stay, prolonged antibiotic therapy and risk of recurrence, which has been reported in 10-20% of patients managed conservatively [6]. The early detection of complex appendicitis has been greatly enhanced by advancements in diagnostic imaging, particularly computed tomography (CT) and ultrasonography enabling better patient selection for either early or delayed surgical intervention. Additionally, the increased use of minimally invasive surgical techniques, particularly laparoscopic appendectomy, has further influenced the management strategies for complicated appendicitis by reducing post-operative pain, wound infection rates and recovery time [7]. Despite numerous studies and meta-analyses, the optimal timing of appendectomy in complicated appendicitis remains controversial. Some surgeons advocate early appendectomy for definitive treatment and reduced overall treatment time, while others recommend delayed appendectomy to minimize operative difficulty and complications. The lack of universal consensus highlights the need for further comparative studies evaluating clinical outcomes, post-operative complications and hospital stay associated with each approach [8]. Therefore, it is of interest to evaluate the relative merits of early and delayed appendectomy in the treatment of severe appendicitis and evaluate operative parameters, post-operative complications and hospital stay in order to determine the most effective management strategy for this common surgical emergency.

## Materials and Methodology:

## Study design:

The current research compared the results of early and delayed appendectomy in individuals with complex appendicitis. The study was designed as a prospective comparative observational study. Comparing the two methods of care allowed the researchers to examine operating parameters, post-operative problems and hospital stay length.

## Study setting:

A tertiary care teaching hospital's Department of General Surgery conducted the research over the course of 18 months. During the trial period, all patients who presented with symptoms and imaging that may indicate a complex appendicitis were evaluated for eligibility.

## Study population:

One hundred individuals with complex appendicitis were a part of the research. Two equal groups of patients were formed according to the treatment plan.

[1] Group A - Early Appendectomy (n = 50): Patients underwent surgical removal of the appendix within 24-48 hours of diagnosis during the same hospital admission.

[2] Group B - Delayed Appendectomy (n = 50): Patients initially received conservative management followed by interval appendectomy after 6-8 weeks once the acute inflammation subsided.

## Inclusion criteria:

Patients fulfilling the following criteria were included in the study:

[1] Patients aged 18-65 years.

[2] Patients diagnosed with complicated appendicitis, including appendicular mass, appendicular abscess, or perforated appendicitis.

[3] Diagnosis confirmed by clinical examination and radiological investigations such as ultrasonography or contrast-enhanced CT scan.

[4] Patients who provided informed consent for participation in the study.

## Exclusion criteria:

The following patients were excluded from the study:

[1] Patients with uncomplicated acute appendicitis.

[2] Patients with generalized peritonitis requiring emergency laparotomy.

[3] Patients with severe comorbid conditions such as uncontrolled diabetes, severe cardiac disease, or coagulopathy.

[4] Pregnant women.

[5] Patients unwilling to participate in the study.

## Diagnostic evaluation:

All patients presenting with suspected complicated appendicitis underwent detailed clinical evaluation including:

[1] History of abdominal pain, fever, vomiting and anorexia

[2] Physical examination focusing on right lower abdominal tenderness or palpable mass

[3] Laboratory investigations including complete blood count, C-reactive protein and serum electrolytes

Radiological confirmation was obtained through ultrasonography of the abdomen or contrast-enhanced CT scan, which helped identify appendicular mass, abscess, or perforation.

## Treatment protocol:

## Group A: Early appendectomy:

Patients in this group underwent early surgical intervention within 24-48 hours of admission. The surgical procedure was performed either by open appendectomy or laparoscopic appendectomy depending on surgeon preference and patient condition. Standard perioperative antibiotic prophylaxis and post-operative care were provided.

## Group B: Delayed appendectomy

Patients in this group were initially managed conservatively using the following measures:

[1] Intravenous broad-spectrum antibiotics

[2] Intravenous fluids and electrolyte correction

[3] Analgesics and antipyretics

[4] Bowel rest and close monitoring

Patients showing clinical improvement were discharged after stabilization and scheduled for interval appendectomy after 6-8 weeks. If the patient failed conservative management or developed worsening symptoms, emergency surgery was performed.

## Study parameters evaluated:

The following parameters were recorded and compared between the two groups:

[1] Demographic variables (age, gender).

[2] Duration of symptoms before admission.

[3] Operative time (in minutes).

4] Intraoperative findings (perforation, abscess, adhesions).

[5] Post-operative complications, including:

1) Wound infection

2) Intra-abdominal abscess

3) Ileus

4) Recurrence of symptoms

[6] Length of hospital stays (in days).

[7] Need for conversion or additional surgical procedures.

## Data collection:

All relevant clinical data were recorded in a pre-designed data collection proforma. Follow-up of patients was performed during hospital stay and during outpatient visits after discharge to evaluate post-operative recovery and complications.

## Statistical analysis:

Statistical Package for the Social Sciences (SPSS) version 22.0 was used for analysis after the data was input into Microsoft Excel.

[1] Age, surgical time and length of hospital stay were quantitative variables that were represented as mean ± standard deviation.

[2] The frequency and percentage were used to convey qualitative factors, such as complications.

[3] We used Student's t-test for continuous variables and Chi-square test for categorical ones to compare the two sets of data.

It was deemed statistically significant if the p-value was less than 0.05.

## Results:

A total of 100 patients diagnosed with complicated appendicitis were included in the study and divided into two groups: Group A: Early Appendectomy (n = 50), Group B: Delayed Appendectomy (n = 50). There was a comparison of the two groups' demographics, surgical results and post-operative progress (Table 1). The average age of the patients who had appendectomy at an earlier stage was 36.8 years, while in the delayed appendectomy group it was 38.1 years, showing no statistically significant difference (p = 0.54). Male patients constituted 60% in the early group and 56% in the delayed group, while females accounted for 40% and 44% respectively, demonstrating comparable gender distribution. The mean WBC count was 13.2 x10^3^/mm^3^ in the early group and 12.9 x10^3^/mm^3^ in the delayed group, with no significant difference (p = 0.63). This indicates that both groups were demographically comparable before intervention. The mean operative time was significantly shorter in the early appendectomy group (64.5 minutes) compared to 72.8 minutes in the delayed appendectomy group (p = 0.003). Additionally, there was a statistically significant difference between the groups in terms of mean intraoperative blood loss: the early surgery group lost 82 ml compared to the delayed group's 95 ml (p = 0.01). Intraoperative adhesions were observed in 36% of early appendectomy patients compared with 56% in delayed appendectomy patients (p = 0.04). The higher incidence of adhesions in delayed surgery may be due to inflammatory fibrosis during the waiting period (Table 2). Although there was no statistically significant difference (p = 0.37), 10% of patients who had their appendix removed early and 16% of patients who had their appendix removed late had post-operative wound infections. Intra-abdominal abscess occurred in 6% of early cases and 8% of delayed cases, also showing no significant difference (p = 0.69). The mean hospital stay was significantly shorter in early appendectomy (4.1 days) compared to 6.8 days in delayed appendectomy (p < 0.001). Additionally, 10% of patients in the delayed appendectomy group experienced recurrent symptoms before surgery, requiring readmission (p = 0.02) (Table 3).

## Discussion:

Complicated appendicitis remains a challenging surgical condition and the optimal timing of appendectomy continues to be debated among surgeons. The present study compared early versus delayed appendectomy in 100 patients, evaluating operative difficulty, post-operative complications and hospital stay. Late appendectomy (72.8 minutes) and early appendectomy (64.5 minutes) had substantially longer mean operating times in the present research. Similar findings showed that early surgical intervention results in reduced operative time and faster recovery [9]. Intraoperative adhesions were observed in 36% of early cases compared with 56% of delayed cases, indicating that delaying surgery may increase operative difficulty due to inflammatory fibrosis. This observation is consistent with similar study which reported increased adhesions and technical difficulty in interval appendectomy cases [10]. Post-operative complications such as wound infection occurred in 10% of early appendectomy patients compared with 16% in delayed appendectomy patients. Although the difference was not statistically significant, previous studies have reported similar trends. A large population-based study involving over 31,000 patients demonstrated higher complication rates and readmissions in delayed surgery compared with early appendectomy [11]. Hamed *et al.* reported that early appendectomy shortens overall hospital stay and reduces costs but is associated with a higher rate of wound infections, whereas delayed surgery lowers the need for major resections and overall complications, highlighting a trade-off that supports individualized timing based on patient risk and disease severity [12]. The present study also demonstrated a significantly shorter hospital stay in the early appendectomy group (4.1 days) compared with the delayed group (6.8 days). Similar findings were reported in a clinical study where early appendectomy reduced hospital stay from 9.7 days to 5.8 days, highlighting the advantage of early surgical intervention [13]. Another important finding of the present study was the 10% recurrence rate in patients managed initially with delayed appendectomy, which required readmission before interval surgery. Recurrence rates of 10-20% following conservative management have also been reported in previous studies evaluating interval appendectomy [14, 15]. Kumar *et al.* found that early appendectomy (≤12 h) was associated with significantly shorter hospital stay and fewer postoperative wound infections than delayed surgery (12-24 h), suggesting that prompt operative management may improve short-term outcomes and reduce resource use in uncomplicated presentations [16]. In addition, research has linked postponed appendectomy to higher healthcare costs, more hospital stays and other negative outcomes and higher readmission rates, reinforcing the benefits of early surgical management in complicated appendicitis [17, 18]. Generally, the current study's findings indicate that appendectomy performed during the first hospitalization offers final therapy. Reduces hospital stay and avoids recurrence during the waiting period. However, both approaches remain safe and effective when carefully selected based on patient condition and surgeon expertise.

## Conclusion:

A shorter operating time, shorter hospital stay and lower recurrence rates were linked with early appendectomy in severe appendicitis compared to delayed appendectomy, according to the current research. While both groups saw similar rates of surgical complications, the delayed appendectomy group had to endure longer treatment durations and was at risk of recurrence before interval surgery. Therefore, early appendectomy appears to be a safe and effective approach for the management of complicated appendicitis and may be preferred in suitable patients to achieve definitive treatment during the initial hospital admission.

## Figures and Tables

**Table 1:** Demographic characteristics of patients

**Parameter**	**Early Appendectomy (n=50)**	**Delayed Appendectomy (n=50)**	**p value**
Mean age (years)	36.8 ±10.2	38.1 ±11.4	0.54
Male	30 (60%)	28 (56%)	0.68
Female	20 (40%)	22 (44%)	
Mean WBC count	13.2 ±3.1	12.9 ±3.4	0.63

**Table 2:** Operative parameters

**Parameter**	**Early Appendectomy**	**Delayed Appendectomy**	**p value**
Mean operative time (minutes)	64.5 ±12.3	72.8 ±14.1	0.003
Mean blood loss (ml)	82 ±20	95 ±24	0.01
Adhesions encountered	18 (36%)	28 (56%)	0.04

**Table 3:** Post-operative outcomes

**Parameter**	**Early Appendectomy**	**Delayed Appendectomy**	**p value**
Wound infection	5 (10%)	8 (16%)	0.37
Intra-abdominal abscess	3 (6%)	4 (8%)	0.69
Mean hospital stay (days)	4.1 ±1.2	6.8 ±1.9	<0.001
Recurrence before surgery	0	5 (10%)	0.02
